# Monitoring optoporated process on mammalian cells by real-time measurement of membrane resealing time

**DOI:** 10.1117/1.JBO.28.6.065006

**Published:** 2023-06-29

**Authors:** Xiaofan Du, Lei Fu, Zhuqu Wang, Zhenxi Zhang, Shudong Jiang, Jing Wang, Cuiping Yao

**Affiliations:** a Xi’an Jiaotong University, Institute of Biomedical Photonics and Sensing, School of Life Science and Technology, Key Laboratory of Biomedical Information Engineering of Ministry of Education, Xi’an, China; b First Affiliated Hospital of Xi’an Jiaotong University, Shaanxi Provincial Center for Regenerative Medicine and Surgical Engineering, Xi’an, China; c Dartmouth College, Thayer School of Engineering, Hanover, New Hampshire, United States; d Norris Cotton Cancer Center, Dartmouth-Hitchcock Medical Center, Lebanon, New Hampshire, United States

**Keywords:** optoporation, resealing time, gold nanoparticle, voltage sensitive dye, intracellular delivery efficiency, Monte Carlo simulation

## Abstract

**Significance:**

Resealing time based loading efficiency of optoporation is the key parameter for drug or gene delivery. This work describes a comparatively simple optical approach to directly measure the cell membrane resealing time of the gold nanoparticle mediated photoporation.

**Aim:**

To establish a membrane potential detection optical system, which can provide a direct measurement of resealing time of the optoporated cells.

**Approach:**

Voltage sensitive dye has been used to label the gold nanoparticle covered cell before laser activation and the resealing time was estimated from the voltage change due to the fluorescence light intensity change before and after laser activation. The approach has been validated by the simulated data based on diffusion model and Monte Carlo simulation and the experimental data obtained from a flow cytometry analysis.

**Results:**

The measured resealing time after perforation varied from 28.6 to 163.8 s on Hela cells when the irradiation fluence was increased, with a correlation coefficient ($R^{2}$) of 0.9938. This result is in agreement with the resealing time (1-2 min) of photothermal porated Hela cells measured by electrical impedance method. The intracellular delivery efficiency of extracellular macromolecular under the same irradiation fluence depends mainly on diffusion velocity rather than pore size.

**Conclusion:**

The method described here can be used to directly measure resealing time of optoporated cells for accurately estimating the loading efficiency on discovering the mechanism of optoporation.

## 1 Introduction

Nanoparticle-mediated pulsed laser induced photoporation has shown great potential in clinical^1^^,^^2^ and pre-clinical^3^ applications since it is a powerful approach to intracellularly deliver membrane-impermeable macromolecules including gene and protein into a target cell.^4^^,^^5^ This photoporation depends less on cell type and can be applied to either a single cell^6^^–^^8^ or a cell group with large numbers of cells.^9^^–^^11^ Among different types of nanoparticles, gold nanoparticle (AuNP)-mediated photoporation has been extensively studied,^12^^–^^17^ and the results have shown that the vapor nanobubbles (VNBs) induced by pulsed laser activated AuNPs are more efficient for intracellular delivery of extracellular materials, also called loading efficiency, compared to that due to the direct heating generated by the photothermal effect.^4^^,^^12^^,^^13^^,^^15^^,^^18^^–^^23^ Recently, VNBs have been applied to destroy vitreous opacities aimed for the treatment of ophthalmologic diseases^24^ and have even been used *in vivo* to perforate the retinal ganglion cells for therapies of retinal degenerative diseases.^25^ In these applications, a critical parameter is the transient membrane pore opening time after irradiation, during which the extracellular molecules can enter into cells before membrane is self-repaired.

To achieve rapid repair and control perforation dynamics of porated cells, the mechanism of cell membrane self-repair, such as how to modulate the membrane resealing dynamics to enhance therapeutic efficiency, has been studied for many years^26^ in which real-time monitoring of the perforated process on mammalian cells has played a very important role. For example, Schneckenburger et al.^6^ observed a tiny black spot by microscopy right after an irradiation of laser on a Chinese hamster ovarian cell which disappeared within about five minutes; Sankin et al.^27^ obtained scanning electron microscopy (SEM) images of porated cell with about 1−2  μm diameter pore in 10 s after irradiation with short pulse laser. However, both studies monitored the process intermittently and it is difficult to continuously capture the whole process from pore formation to total resealing. In recent studies, Shirakashi et al.^28^ measured the resealing time using a fluorescent dye of propidium iodide (PI) as a reporter through flow cytometry. They estimated the resealing time using a model based on the assumption that the permeability of PI decreases exponentially with time during resealing the plasma membrane’s permeability to PI. The resealing time constant of the electroporated mammalian cell membranes was reported to be in the range of 200 to 300 s.^28^ Davis et al.^29^ used a similar method to estimate the resealing time and pore size of femtosecond laser induced perforated cells, and the small difference is that they monitored the efflux of a small intracellular dye after photoporation rather than flux of dye into cells. Since the resealing time in these studies was estimated with the model based on single cell, some disagreement occurred between experimental and simulated data. Yamane et al.^30^ first measured the resealing time of photothermal porated mammalian cells. However, the design and fabrication of a complex metallic microdisk-integrated electrical impedance sensor was necessary for perforating cells.

In this work, to address the issues mentioned earlier, a comparatively simple optical approach has been utilized to directly measure the cell membrane resealing time of the gold nanoparticle mediated photoporation. In this approach, since the membrane potential changes after photoporation and returns to the resting potential with the membrane resealing, the photoporation process and membrane resealing time could be monitored and obtained by continuously measuring the membrane potential change with voltage sensitive dye after photoporation. Furthermore, a Brownian motion diffusion-based model has been used to estimate the loading efficiency as a function of the obtained membrane resealing time and the delivered molecule weight (MW) by Monte Carlo simulation. Our results have shown that the resealing time is in the range of 28.6 to 163.8 s, corresponding to the different irradiation fluence. The coefficient of determination ($R^{2}$) of the fitting curve of loading efficiency versus different irradiation fluence were 0.9604 and 0.9944, loading efficiency versus MW were 0.9891 and 0.9918 in experimental and simulated data, respectively. Furthermore, the $R^{2}$ values between the simulated and experimental data were 0.9251 and 0.9618 for laser fluence and MW, respectively. This work provides a direct measurement method for determining the resealing time of optoporated cells.

## 2 Materials and Methods

### 2.1 Materials and Reagents

Hela cells were obtained from cell bank of Chinese Academy of Sciences (Shanghai, China). AuNPs were purchased from BBI solution (United Kingdom). Fluorescein isothiocyanate-dextran (FITC-D) with MW of 5 kDa, 10 kDa, 40 kDa, 70 kDa and 100 kDa was purchased from Xi’an ruixi Biological Technology Co., Ltd (Xi’an, China). PI and penicillin/streptomycin were purchased from Solarbio Life Sciences, Ltd. (Beijing, China). Dulbecco’s modified eagle medium (DMED, Hyclone, United States), phosphate buffer saline (PBS, Hyclone, United States) and trypsin (Hyclone, United States) were purchased from the agent Xi’an Haimeng Experimental Technology Co., Ltd. (Xi’an, China). Fetal bovine serum (FBS, Gibico) and voltage sensitive dye Di-4-ANEPPS (Invitrogen) were obtained from ThermoFisher Scientific. Dimethyl sulfoxide was obtained from Sigma-Aldrich (United States).

### 2.2 Cell Culture

Hela cells were cultured in DMEM containing 10% FBS supplemented with 1% penicillin/streptomycin at 37°C and 5% ${\mathrm{CO}}_{2}$ atmosphere. Before experiments, cells were plated into a 96-well plate with a density of $0.8\times 10^{4}\text{ cells}$ per well and incubated for 12 h.

### 2.3 Monitoring Membrane Potential Change

**Fig. 1:**
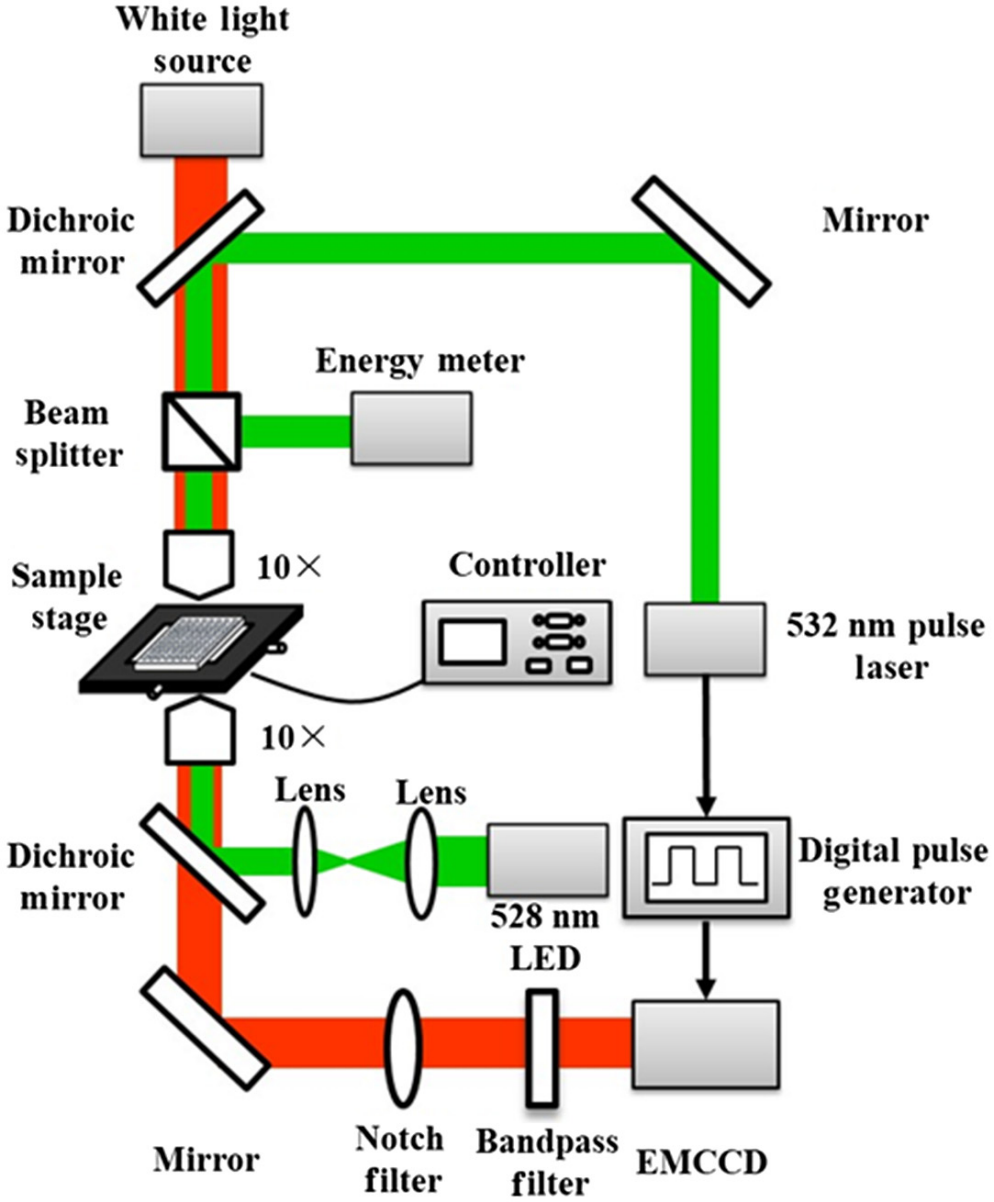
Schematic of photoporation and membrane potential detection system.

A custom-made photoporation experimental system is shown in Fig. 1. The 532 nm pulsed laser beam (Q-smart 450, Quantel, France) was split into two parts by a beam splitter with a split ratio of 10: 90. One part was focused by a 10 × objective (GCO-2131, Daheng Optics, China) to irradiate cells, and the other part was recorded by energy meter (PE50-DIF-C, Ophir Optronics, Israel). The LED with the wavelength of 528 nm and a power of 3 W (GCI-060403, Daheng Optics, China) was used to excite voltage sensitive dye which was added in culture medium with a concentration of 20  μg/ml and washed with PBS after 1 hour incubation. Before the experiment, AuNPs solution with an approximate optical density (OD) of 0.025 was added to cell and incubated for 1 h, and then it was washed with PBS to remove the free AuNPs. A cooled electron-multiplying charge coupled devices (EMCCD) was used to collect the fluorescence images with an exposure time of 1 s. Before irradiating the cell, 100 frame fluorescence images were collected as background images with an interval of 1 s. Then, a 6 ns laser pulse was generated to irradiate cells and 380 frame fluorescence images were obtained after irradiating the cell. A digital delay generator DG645 (Stanford Research Systems Inc., United States) was used to synchronize laser and EMCCD (Xion3, Andor, United Kingdom). A Matlab (Matlab R2020a, MathWorks, United States) program was written for handling the fluorescence and automated graphing the changing curve of relative fluorescence intensity in response to changes in membrane potential. The resealing time was defined as the time point that the relative intensity of membrane potential back up to 90% of resting fluorescence intensity after laser treatment. Then, the resealing time of the pore can be calculated and a function relationship between resealing time and laser fluence was established.

### 2.4 Photoporation on Hela Cells

The same system shown in Fig. 1 was used for photoporation. A 532 nm 6 ns pulse laser (Q-smart 450, Quantel, France) was employed to irradiate the cell with a repetition frequency of 10 Hz, the laser beam had a Gaussian profile with a radius of 356  μm at the interface with the cell. Meanwhile, the two-dimension motion stage (MC600, Zolix Instruments Co., Ltd, China) carrying the 96-well moved line by line at a velocity of 1  mm/s. The distance between two lines was 0.1 mm. Before laser treatment, 60 nm AuNPs solution with OD of 0.025 was added into cell medium and incubated for 1 h and it attached on cell membrane by cell adhesion effect. After that, PBS was used to wash the free AuNPs out. Then, fresh culture medium mixed with FITC-D (0.4  mg/ml) was supplemented into the well and the cell was treated with the system. Afterwards, cells were cultured in humidified incubator for 30 min, and then washed with PBS. Afterward, fresh culture medium mixed with PI (1  μg/ml) was supplemented into the well and washed with PBS after 10 min. Then, the fluorescence image was obtained by a fluorescence microscope (ECLIPSE TI, Nikon, Japan), and the perforation efficiency was quantitatively evaluated with a FACScan flow cytometer (BD Biosciences, United States). As shown in Fig. S1 in the Supplementary Material, the cells located in Q1 were defined as perforated cells, and the percentage of Q1 was defined as loading efficiency. The cells located in Q2 and Q3 were defined as dead cells, and the percentage of Q2 and Q3 was defined as death rate. The permeability and death rate of photoporated cells was estimated as descripted in our previous publication.^31^

### 2.5 Model of Loading Efficiency Based on Brownian Motion

To estimate the probability that the foreign fluorescence molecule of FITC-D entered into the cell, a model based on Brownian motion was established by Monte Carlo simulation.

In the simulation, the cell was set as a ball in three-dimensional space coordinate, marks was appeared randomly on the surface that indicated pores on the cell membrane. Each of foreign materials was treated as an independent point without any associated area. The point of foreign materials was randomly generated and dispersed at t=0. Each of the foreign material randomly moved obeying Brownian motion, and the moving step was determined according to MW of foreign materials. The probability that each point diffused into the coordinate of pore was defined as delivery efficiency.

According to Brownian motion materials size determined the diffusion velocity of materials, it was quantified by the step size of movement in our program. The relationship between moving velocity and material size was deduced as follow: the foreign materials randomly diffused in culture medium with the manner of Brownian motion, and the diffusion coefficient can be determined as the following equation:

$$D=\frac{k_{B}T}{6\pi \eta R},$$ (1)

where $k_{B}$ is Boltzmann constant, T is the Temperature, η is the fluid viscosity, and R is the radius of foreign materials. According to the Einstein viscosity relation, the viscosity can be depicted as the following equation:

$$\eta =\frac{2.5NV_{e}}{M},$$ (2)

where N is Avogadro’s number, $V_{e}$ is volume of an equivalent spherical particle, and M is the molecular weight of foreign materials. Since $V_{e}=4/3\pi R^{3}$, the Eq. (2) can be rearranged as follows:

$$R=\sqrt[{3}]{\frac{3\eta M}{10\pi N}}.$$ (3)

After pulse laser irradiating on gold nanoparticle, the temperature of gold nanoparticle and surrounding medium reached to steady state in several nanoseconds.^32^ Therefore, we deem that the temperature was constant in the process that foreign materials entered into the cell. In this work, the concentration of foreign materials was constant and there was little difference between the MW of foreign materials used in experiments. So, we assumed the viscosity was constant. Thus, we can conclude that diffusion coefficient was inversely proportional to the radius of foreign materials from Eq. (1), and the radius of foreign materials was proportional to the cube root of MW from Eq. (3). In a certain interval of time, the velocity of foreign molecule was proportional to diffusion coefficient.^33^ Therefore, we can conclude the following equation:

$$V\propto D\propto \frac{1}{R}\propto \frac{1}{\sqrt[{3}]{M}},$$ (4)

where V was the Brownian motion velocity of foreign materials. Thus, the parameter of moving velocity of foreign materials was set according to Eq. (4) in program. The program of this model was performed in Matlab.

## 3 Results

### 3.1 Measurement of Cell Membrane Resealing Time

**Fig. 2:**
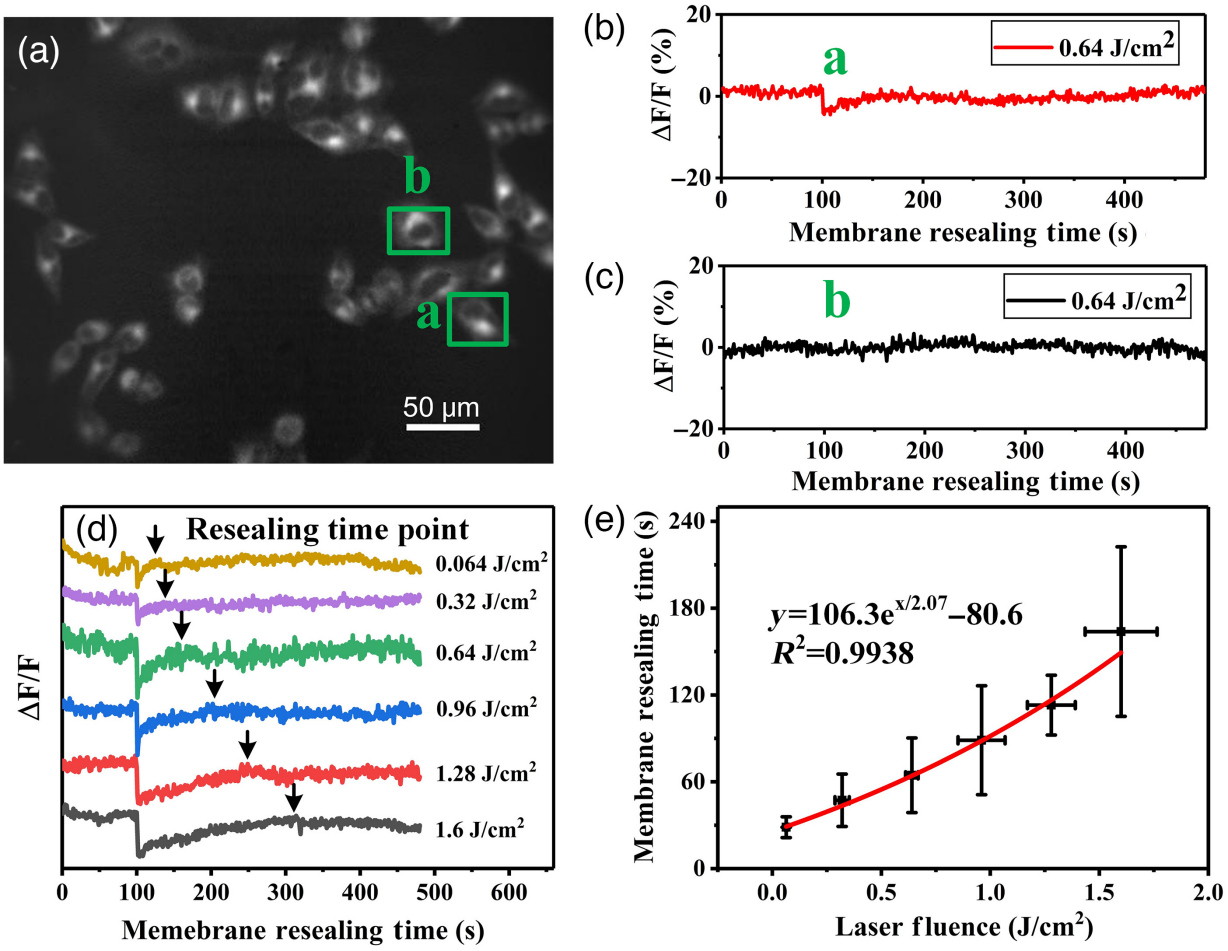
Establishing the function relationship between laser fluence and resealing time. (a) The fluorescence image of Hela cells stained by Di-4-ANEPPS after laser treatment; green boxes labeled “a” and “b” show a representative perforated and unperforated cell, respectively. Relative fluorescence intensity (ΔF/F) of (b) a perforated cell [marked as “a” in panel (a)] and (c) an unperforated cell [marked as “b” in panel (a)], in response to a change in membrane voltage with time, respectively. (d) Relative fluorescence (ΔF/F) intensity in response to a change in membrane voltage with time after cell was treated with different laser fluence. The laser fluence was in the range of 0.064 to $1.6\;J/{\mathrm{cm}}^{2}$. (e) The relationship between laser fluence and resealing time. The error bars show the ranges of laser fluence and membrane resealing time from at least ten independent measurements; the red line is the fitted line, which can be expressed as $y=106.{3e}^{x/2.7}-80.6$. Ten cells from different independent experiments (n≥3) were selected to reduce error for each laser fluence.

Cell membrane resealing time can be obtained by monitoring the relative fluorescence intensity (ΔF/F), which changes in respond to the membrane voltage changes. ΔF/F denotes the change in fluorescence intensity relative to resting fluorescence intensity. The fluorescence image of cells [Fig. 2(a)] was captured by EMCCD in a custom-made experimental system (Fig. 1). We captured 100 frames of background firstly and 380 frames of fluorescence images after one pulse of laser irradiation at imaging speed of 1 frame/second. The background images were used as calibration. Considering the laser irradiation for photoporation was only last 6 ns (laser pulse width), the fluorescence photobleaching was mainly due to the LED. A linear correction method was used to reduce the effect of photobleaching (Fig. S2 in the Supplementary Material). Figure 2(a) is an example of the fluorescence images captured after the laser treatment. Figures 2(b) and 2(c) were the relative fluorescence intensity of a perforated and an unperforated cell [marked as a and b in Fig. 2(a)] over time, respectively, corresponding to a change in membrane voltage with time. It can be seen clearly that when the laser pulse turned on at ∼100  s, there is a dip appeared on fluorescence intensity from the cell membrane of the perforated cell [Fig. 2(b) and Fig. S2 in the Supplementary Material], whereas no significant change can be observed on that come from the unperforated cell [Fig. 2(c)]. This dip in fluorescence intensity can be attributed to the formation of a pore on the cell membrane after laser irradiation. Subsequently, the pore underwent recovery once the irradiation ceased. The presence of the pore disrupted the static potential of the cell membrane and influenced the curve representing the relative fluorescence intensity of the membrane. As showed in Fig. 2(d), the resealing time was defined as the time from laser on (100’th seconds) to relative fluorescence intensity recovering to 90% of resting intensity,^34^ and a computer program was coded to determine the resealing time. It increased while the irradiation fluence increased and the increasing rate can be defined by the function of $y=106.{3e}^{x/2.7}-80.6$ [Fig. 2(e)].

### 3.2 Evaluation of Cell Photoporation

FITC-D with a MW of 10 kDa (FD10) was employed as foreign material to study the VNBs induced photoporation. 60 nm gold nano-sphere particles with an approximate OD of 0.025 was chosen to assist photoporation because 60 nm is the optimizing diameter for vapor bubble generation according our previous research.^35^^,^^36^ Cells were irradiated by different fluence at 0.064, 0.32, 0.64, 0.96, 1.28, and $1.6\;J/{\mathrm{cm}}^{2}$, respectively.

**Fig. 3:**
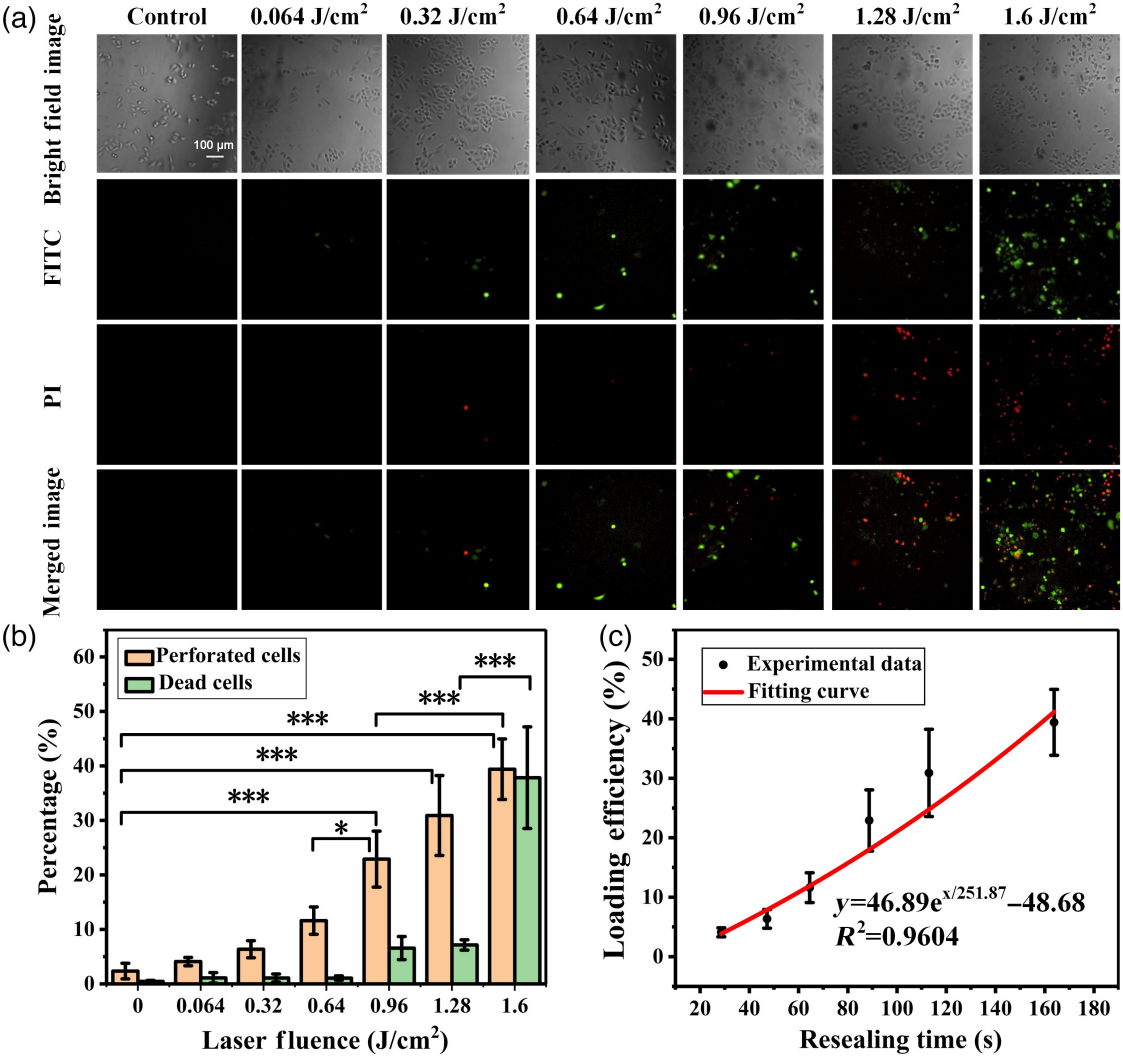
Delivering the FD10 into Hela cells by VNBs induced photoporation. (a) Fluorescence images of intracellular delivery of FD10 after treated with different laser fluence; FITC, fluorescein isothiocyanate and PI, propidium iodide. (b) The percentage of perforated cells and dead cells evaluated by flow cytometry at different laser fluence. Perforated cells were the FITC positive and PI negative cells. Dead cells were the PI positive cells. Statistically significant differences were indicated by *** (p<0.001) and * (p<0.05). Error bars represent standard deviation from the mean of independent experiments (n=3). (c) The loading efficiency as the function of resealing time by using experimental data. The error bars show the range of loading efficiency from the mean of independent experiments (n=3); the red line is the fitted line, which can be expressed as $y=46.{89e}^{x/251.87}-48.68$.

Fluorescence images of laser treated cells are shown in Fig. 3(a), and the quantitative statistic results are shown in Fig. 3(b) where 5000 cells are analyzed (Fig. S1 in the Supplementary Material). The results demonstrated a significant increase in loading efficiency when using laser fluence levels above $0.96\;J/{\mathrm{cm}}^{2}$. It can reach to 39.4% at $1.6\;J/{\mathrm{cm}}^{2}$; however, at this point, the death rate was significantly increased to 37.83%. Based on the loading efficiency of experimental data and the relationship between resealing time and laser fluence, the loading efficiency as a function of resealing time was achieved [Fig. 3(c)].

**Fig. 4:**
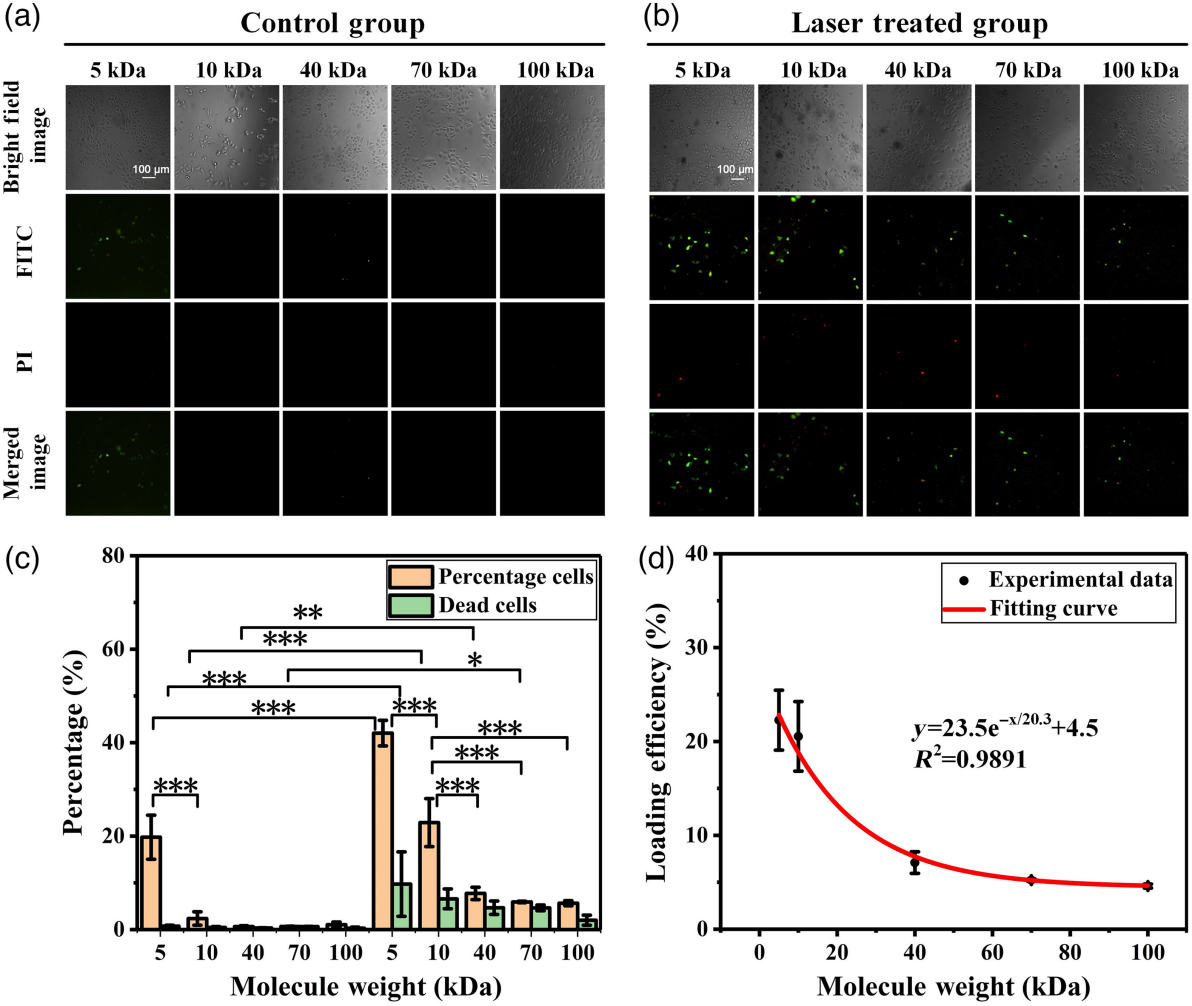
Delivering the FITC-D with different MWs (5 kDa, 10 kDa, 40 kDa, 70 kDa, and 100 kDa) into Hela cells by VNBs induced photoporation. (a) Fluorescence images of the control group, cells were incubated with AuNPs, and FITC-D and PI were added for imaging. (b) Fluorescence images of the treated group (laser fluence: $0.96\;J/{\mathrm{cm}}^{2}$). (c) The percentage of perforated cells and dead cells determined by flow cytometry (laser fluence: $0.96\;J/{\mathrm{cm}}^{2}$). Perforated cells were the FITC positive and PI negative cells. Dead cells were the PI positive cells. Statistically significant differences were indicated by *** (p<0.001), ** (p<0.01), and * (p<0.05). Error bars represent standard deviation from the mean of independent experiments (n=3). (d) The loading efficiency as the function of MW using experimental data. The error bar shows the range of loading efficiency from the mean of independent experiments (n=3); the red line is the fitted line, which can be expressed as $y=23.{5e}^{-x/20.3}+4.5$.

To study the impact of foreign material size on perforation efficiency, we irradiated the cells with different size of molecules, under certain laser fluence. Laser fluence of $0.96\;J/{\mathrm{cm}}^{2}$ was selected for its higher delivery efficiency and lower death ratio with minimal cell disturbance (i.e., cell deformed under bright field) compared to that under other laser fluence. FITC-Ds with different MWs of 5 kDa, 10 kDa, 40 kDa, 70 kDa, and 100 kDa were used in the experiments. The fluorescence images and the quantitative statistical results were shown in Figs. 4(a), 4(b), and 4(c), respectively. It clearly showed that FITC-Ds with small MW were more easily to be delivered into cells than that of large MW. The perforation efficiency of 5 kDa can reach to 42.03% at $0.96\;J/{\mathrm{cm}}^{2}$, while it was 19.76% in the control group without laser treatment indicating some of the FITC-D (5 kDa) were entered into cell by endocytosis. Indeed, the percentage of perforated cells was significant decreased compared to that under 5 kDa when MW of FITC-Ds was greater than or equal to 10 kDa in control groups. Similarly, the loading efficiency of VNBs induced photoporation was decreased while increasing MW. Based on the loading efficiency of experimental data, the loading efficiency as a function of MW was achieved [Fig. 4(d)].

### 3.3 Estimating the Delivery Efficiency of Photoporation

**Fig. 5:**
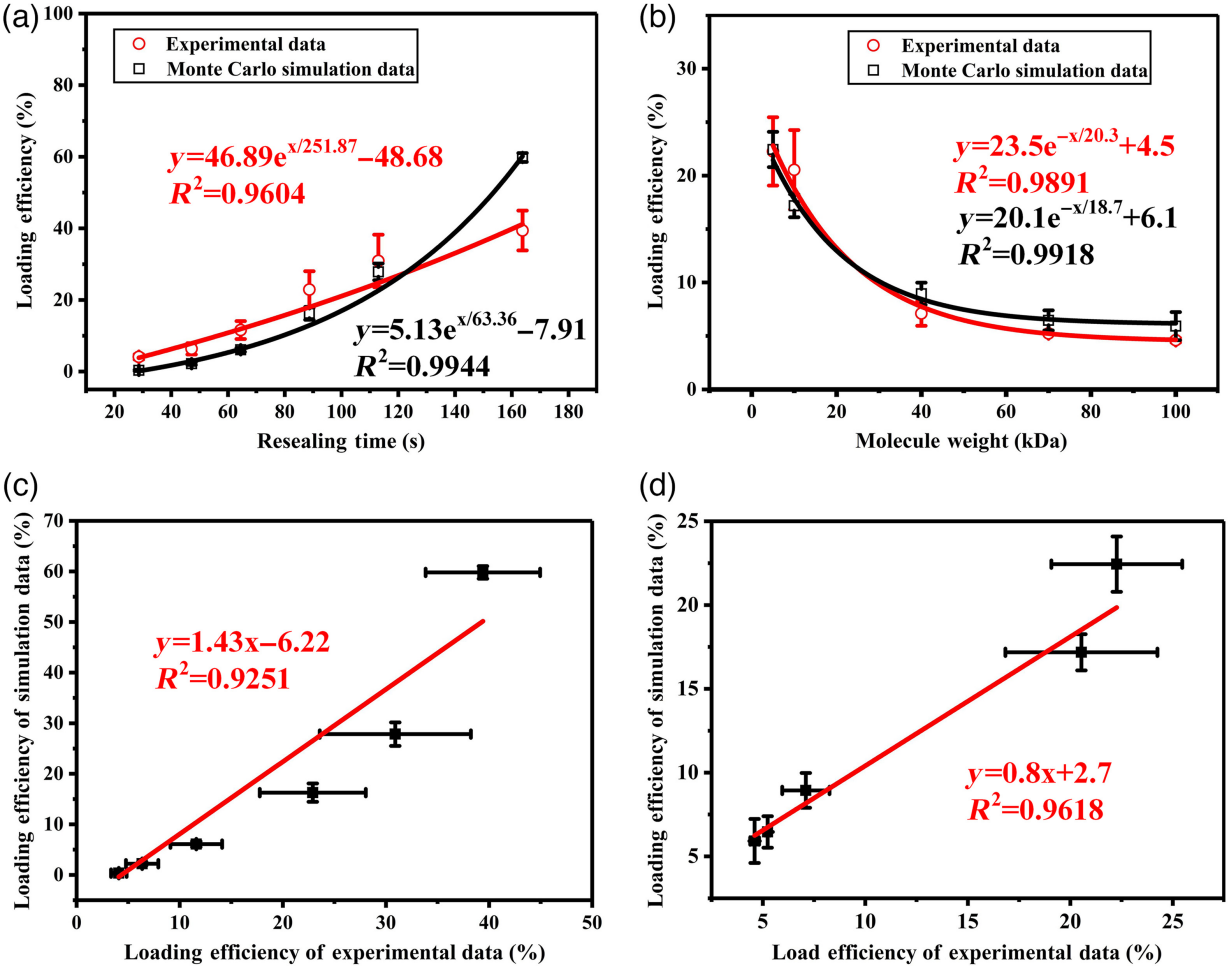
Comparison of loading efficiencies between experimental and simulated data. (a) The loading efficiencies as the functions of resealing time using experimental and simulated data, respectively. (b) The loading efficiency as a function of MW using experimental and simulated data, respectively. (c) Simulated versus measured loading efficiency at different resealing time. (d) Simulated versus measured loading efficiency for different MW. The red line is the fitted line of experimental data, and the black line is the fitted line of simulated data in panels (a) and (b); the red circles are the experimental data and the black squares are the simulated data in panels (a) and (b); and the red line is the fitted line in panels (c) and (d). Error bars represent standard deviation from the mean of independent experiments (n=3) for experimental data and the mean of independent simulation experiments (n=10) for simulation data.

To find out the relationship between loading efficiency and resealing time, a diffusion-based model was established to predict the loading efficiency and the dynamic process that extracellular materials diffusing into cell is shown in Video 1. FITC-Ds with a MW of 10 kDa was used as a model (of foreign materials) to study the loading efficiency changes due to the certain pore resealing time (opening time) that determined by corresponding laser fluence according to the function relationship in Fig. 2(e). Based on the diffusion-based model (see Sec. 2), the loading efficiency as a function of resealing time can be achieved with a constant MW of exogenous molecules [Fig. 5(a)]. The experimental and simulated results were presented an exponential growth while increasing the resealing time within a range of 28.6 to 163.8 s. The $R^{2}$ were 0.9604 and 0.9944 for experimental and simulated data, respectively. Based on the above results (Fig. 4), we can see that the molecular weight/size is another important parameter for loading efficiency. FITC-Ds with different MW (5 kDa, 10 kDa, 40 kDa, 70 kDa, and 100 kDa) and constant resealing time were also implemented into the model. When using the loading efficiency corresponding to different MW at $0.96\;J/{\mathrm{cm}}^{2}$ (obtained from the previous experiments), the loading efficiency as a function of MW can be achieved [Fig. 5(b)]. The $R^{2}$ were 0.9891 and 0.9918 for experimental and simulated data, respectively. It can be seen that the loading efficiency has a downward trend when the molecular weight was increased. To evaluate the accuracy of simulation data, the comparison between the loading efficiency of simulated data and the experimental data were presented in Figs. 5(c) and 5(d). It clearly showed that the loading efficiency of simulation data has a consistent variation trend with the loading efficiency of experimental data.

## 4 Discussion and Conclusion

In this paper, the membrane resealing time of gold nanoparticles mediated cell photoporation activated by nanosecond laser irradiation was directly measured by monitoring the membrane potential changes. A diffusion-based model that can be used to estimate the delivery efficiency of extracellular materials during the resealing time and the corresponding pore size, was developed and validated by comparing the simulated data with experimental results. The results showed that the simulated data is in good agreement with our experimental data, which confirmed that the direct measurement of resealing time by optical method is feasible. In addition, the resealing time-based diffusion model developed in this work can be used to predict the loading efficiency for future clinical applications.

By carefully measuring the membrane potential changes of cells throughout the entire opto-perforation process, we quantified the resealing time directly by measuring the cell membrane potential changes before and after laser irradiation. In this case, the resealing time increased with laser energy in a range of 28.6 to 163.8 s. For the optimal delivery efficiency at the fluence of $0.96\;J/{\mathrm{cm}}^{2}$, the resealing time is about 88.7 s, which is almost as same as the resealing time of photothermal porated Hela cells measured by electrical impedance method.^30^ However, it should be noted that the later method is very time-consuming since it requires the cells seed on the electrodes and have to be measured one by one. Moreover, the electrical impedance is easy to be interfered by environment and the accuracy of the measurement is hard to be maintained.

As one of the indirect resealing time evaluation method, Davis et al. and Shirakashi et al. developed an approach to monitor the efflux or flux of a small intracellular dye after laser irradiation. This approach based on an assumption that during resealing process, the permeability of the plasma membrane to fluorescence dye decreases exponentially with time, and the permeability could be estimated through a complicated model fitting process using measured temporal fluorescence intensity change.^28^^,^^29^ According to their results, the resealing time were in the ranges of 10 to 100 s and 190 to 290 s for Chinese hamster ovarian cells and mouse myeloma cells, respectively. We believe that the large time difference (up to couple of hundreds seconds) compared to our findings may result from the difference of the mechanisms of membrane perforation, pore size, cell type, and environmental factors.^31^ Also, according to He et al., it is reasonable that the viable cell membrane resealing occurring within seconds to minutes.^26^

From our results, the resealing time increased with the increase of the irradiation fluence. Although we have not measured the pore size on the membrane, according to Davis and his colleagues, the laser-induced pore radii increased with increasing laser energy.^29^ It can be concluded that the longer resealing time meant the bigger pore size. It is also confirmed by other studies.^26^ It is in contradiction with the results of the Davis’s study.^29^ In their study, they used decay constant for the concentration difference as a significant portion of the resealing time. Figure 2(e) showed that the relationship between laser fluence and resealing time fit the exponential curve, the reason is that resealing time of cell membrane is proportionable to the pore size^37^ and the pore size is exponentially increased by laser fluence.^38^ In addition, photoporation is mainly induced by vapour nanobubbles here and our previous research has shown that the probability of vapour nanobubble generation has an exponential increasing with laser fluence.^35^ Therefore, the relationship between resealing time and laser fluence can be fitted in an exponential curve.

Also, our experiment results showed that the loading efficiency increased with the increase of irradiation fluence. We assumed that molecules enter into the cells via diffusion, for same molecules with same diffusion velocity, more resealing time meant more molecules entry into cells, which is in agreement with our experiment results (Fig. 3). Since diffusion velocity of material is inversely proportional to the cube root of MW, small molecule with high diffusion velocity enter into cells more easily than big molecule in same resealing time, which is also in agreement with our experiment results (Fig. 4). In Figs. 3(c), and 4(d), the loading efficiency increased when resealing time increased, and decreased when molecular weight increased. We used Monte Carlo simulation to estimate the outcome of an uncertain event to assess the loading efficiency under certain conditions. As shown in Figs. 5(a) and 5(b), these simulation showed that the exponential function was the most fitted relationship for both cases, compared to the other functions. This has been confirmed by our experimental results as well.

For predicting the loading efficiency of cells by resealing time, the diffusion-based model was used because diffusion is the dominant driving force of solute transport through the pore.^29^ The reason of using Monte Carlo simulation to imitate the behavior of molecules was based on Brownian motion is a random movement of fluid molecules (particles). Since the resealing time is the most important parameter for loading efficiency, and the resealing time is corresponding to pore size, we simulated the loading efficiency according to the different resealing times and pore size, which changes following resealing time. The results showed that the simulated curve is in good agreement with the experimental data, which confirmed that it is reasonable to predict loading efficiency using resealing time with diffusion model by Monte Carlo simulation.

However, the loading efficiency of simulated data gradually deviated the experimental data when the resealing time was great than 113 s [Fig. 5(a)]. This difference attributed to death rate induced by long resealing time. Therefore, the model was not accurately to predict the data with long resealing time and high cell death rate. We further studied the relationship between simulated data and experimental data in short resealing time from 28.6 to 113 s. It showed that the predicted loading efficiency was in good agreement with the experimental results (Fig. S3 in the Supplementary Material). Therefore, our diffusion model was suitable to predict loading efficiency under the condition of short resealing time and low cell death rate.

By measuring the resealing time directly through convenient optical method, the mechanism of the gold nanoparticle aided photoporation may be further explored. Through resealing time the diffusion-based model was built to estimate the loading efficiency with Monte Carlo method. The agreement of the experiment results and the simulation verified the measurement precision. And the diffusion-based model is an ideal approach to predict loading efficiency of photoporated cells, the results showed resealing time can predict loading efficiency well.

In this study, Hela cells were used, the optimal loading efficiency was about 22.9% at energy density of $0.96\;J/{\mathrm{cm}}^{2}$ for 10 kDa molecule. Since the delivery efficiency and cell death rate after photoporation depend on resealing of membrane after irradiation, and the resealing time is crucial for cell viability,^29^ the higher death rate of cells at $1.6\;J/{\mathrm{cm}}^{2}$ might due to some cells’ membrane are destroyed permanently (Fig. S4 in the Supplementary Material). Here, gold nanoparticles were attached to cell surface nonspecifically, and the cell death rate increased rapidly while the irradiation energy density was increased. Indeed, in our previous work, much higher loading efficiency of about 70% and lower death rate of 17.5% were achieved in antibody-targeted and trypsinized adherent cells,^39^ suggesting that higher efficiency can be achieved by combining optimized laser parameters with better cell targeting.

Although the optical recording to monitor the resealing time was easy to implement and monitor a population of cells simultaneously, it requires signal-to-noise ratio and collect multi-frame images to correct the baseline shift caused by fluorescence bleaching. More sensitive and stable voltage sensitive dye will improve the accuracy on estimating the resealing time.

## Code, Data, and Materials Availability

Please contact the authors regarding availability of the code and data used in this study. See Supplementary Material for supporting content. The source code is available on Code Ocean at https://doi.org/10.24433/CO.3833662.v1.
