# Supplementary material for: Monitoring optoporated process on mammalian cells by real-time measurement of membrane resealing time
Source: J Biomed Opt. 2023 Jun 29;28(6):065006. doi: 10.1117/1.JBO.28.6.065006 (PMC10308995; doi:10.1117/1.JBO.28.6.065006)
Supplement: Supplementary file 1 [file JBO_028_065006_SD001.docx]

Monitoring photoporated process on mammalian cells by real-time measurement of membrane resealing time

**Xiaofan Du**^a,b,^**^#^, Lei Fu**^a,^**^#^, Zhuqu Wang**^a^**, Zhenxi Zhang**^a^**, Shudong Jiang**^c^**, Jing Wang**^a,^**^*^, Cuiping Yao**^a,^**^*^**

**a**Key Laboratory of Biomedical Information Engineering of Ministry of Education, Institute of Biomedical Photonics and Sensing, School of Life Science and Technology, Xi’an Jiaotong University, Xi’an, Shaanxi 710049, China.

**b**Shaanxi Provincial Center for Regenerative Medicine and Surgical Engineering, First Affiliated Hospital of Xi’an Jiaotong University, Xi’an, Shaanxi 710061, China

cThayer School of Engineering, Dartmouth College, Hanover, New Hampshire 03755, United States; Norris Cotton Cancer Center, Dartmouth-Hitchcock Medical Center, Lebanon, New Hampshire 03766, United States.

.

**Keywords:** Optoporation, Resealing time measurement, Prediction, Monte Carlo method

*Address all correspondence to Cuiping Yao**,** [zsycp@xjtu.edu.cn](mailto:zsycp@xjtu.edu.cn); Jing Wang, [wangjing@xjtu.edu.cn](mailto:wangjing@xjtu.edu.cn)

#These authors contributed equally to this work.

**This file includes:**

Fig. S1 to S4

Legends for Visualization S1


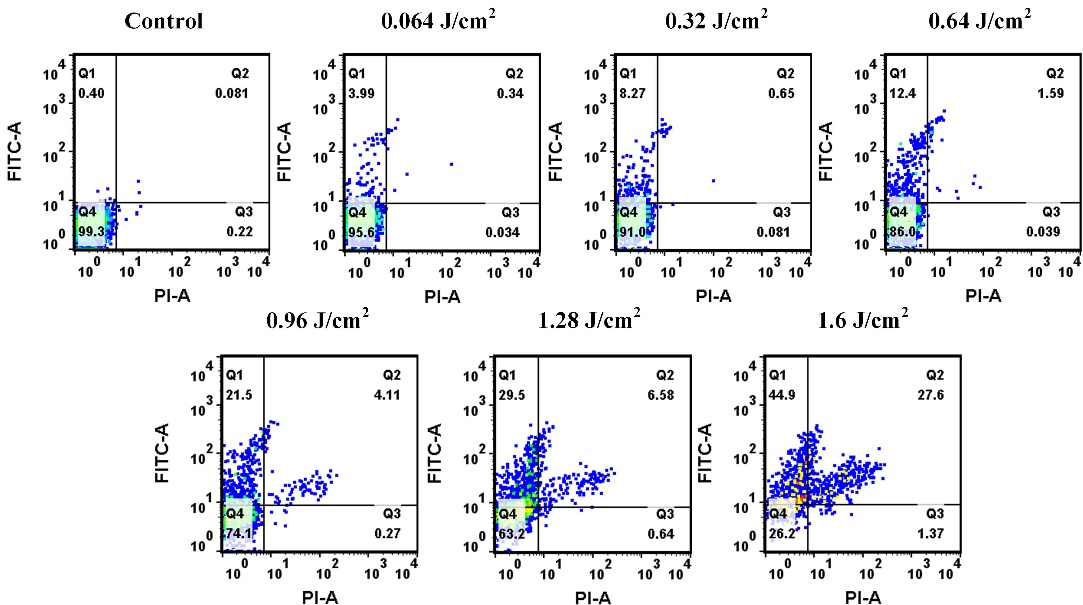


Figure S1. Perforated cells (Q1), dead cells (Q2 and Q3) and normal cells (Q4) were evaluated by flow cytometry. Q1: Fitc+, PI-; Q2: Fitc+, PI+; Q3: Fitc-, PI+; Q4: Fitc-, PI-.


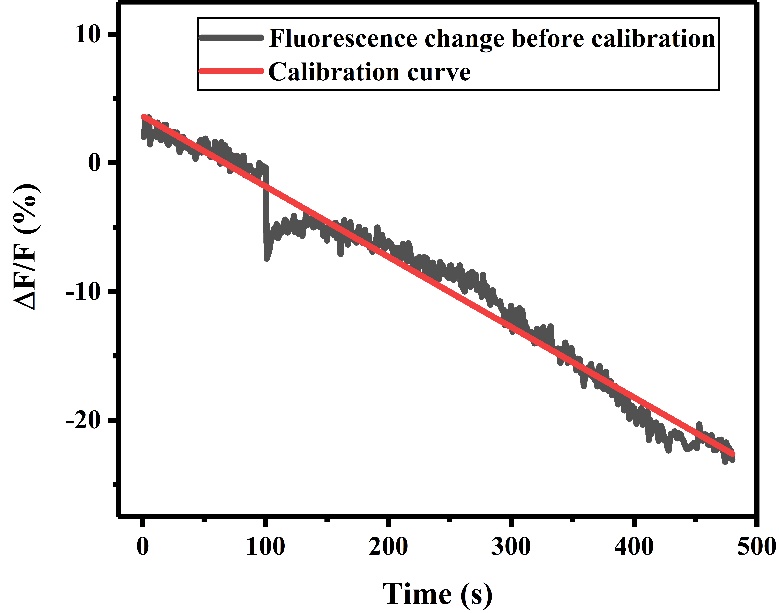


Figure S2. The linear correction of relative fluorescence (∆F/F) intensity in response to a change in membrane voltage, the laser fluence was 0.96 J/cm^2^ and LED fluence was 3 W.


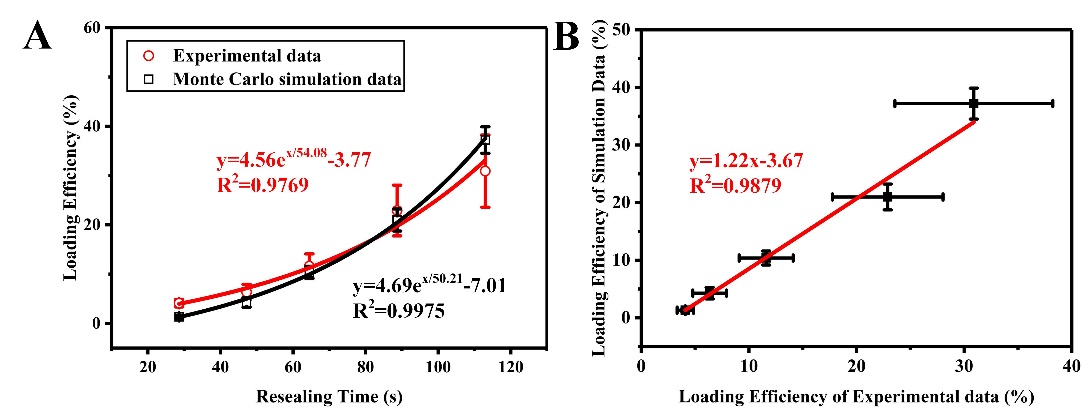


**Figure S3.** Comparison of loading efficiencies between experimental and simulated data. (A) The loading efficiencies as the functions of resealing time by using experimental and simulated data in a range from 28.6 seconds to 113 seconds, respectively. (B) The comparison of the loading efficiency of simulation data on closeness of loading efficiency of experimental data for different resealing time. Error bars represent standard deviation from the mean (n=3) for experimental data and the mean (n=10) for simulation data.


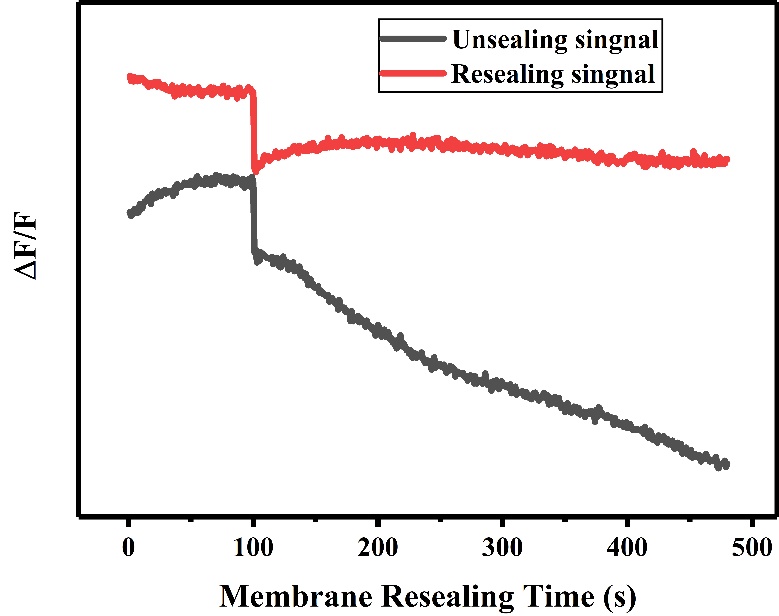


Figure S4. Relative fluorescence intensity in response to a change in membrane voltage with time after treatment by the laser fluence of 1.6 J/cm^2^. Red and black lines represent the relative fluorescence intensity from a perforated or a dead cell, respectively

Legends for Visualization S1

Visualization S1 Description: The simulation of extracellular materials diffusing into cells, red dots represent the materials suspending in the culture medium, green dots represent the materials diffusing into the cell.
